# α6β4 Integrin Genetic Variations (A380T and R1281W) and Breast Cancer Risk in an Argentinian Population

**DOI:** 10.3390/ijms17101540

**Published:** 2016-10-18

**Authors:** Karina Beatriz Acosta, Melina Noelia Lorenzini Campos, Susana Beatriz Etcheverry, Pedro Dario Zapata

**Affiliations:** 1 Instituto de Biotecnología Misiones “Dra. Maria EbeReca” (InBioMis), Facultad de Ciencias Exactas, Químicas y Naturales, Universidad Nacional de Misiones, Ruta Nacional Nº12 km 7 ½, Posadas 3300, Argentina; melinalorenzini@hotmail.com (M.N.L.C.); pdr_dario@yahoo.com (P.D.Z.); 2 Cátedra de Bioquímica Patológica, Facultad de Ciencias Exactas, Universidad Nacional de La Plata, 47 y 115, La Plata 1900, Argentina; etcheverry@biol.unlp.edu.ar; 3 Centro de Química Inorgánica (CEQUINOR, CONICET), Facultad de Ciencias Exactas, Universidad Nacional de La Plata, 47 y 115, La Plata 1900, Argentina

**Keywords:** breast cancer, α6β4 integrin, genetic variations

## Abstract

The α6β4 integrin is composed of the α6 and β4 subunits that are encoded by the *ITGα6* and the *ITGβ4* genes, respectively. The α6β4 main function is to intervene in lamination and epithelia integrity maintenance by cell-matrix interactions. This integrin appears to have importance in breast cancer malignancy, as well as other epithelial carcinomas. The aim of this work was to investigate the potential role of *ITGα6* (A380T) and *ITGβ4* (R1281W) genetic variations in breast cancer susceptibility, in a female population from the northeast region of Argentina (Misiones). We performed a case-control study of 85 breast cancer patients and 113 cancer-free controls. Genotyping was performed by RFLP-PCR. For *ITGα6* (A380T) single nucleotide polymorphism, a high frequency of heterozygous genotype GA in cases compared to controls was observed, achieving values of 48% and 49%, respectively. No association between the A380T SNP and breast cancer development was found (Odds Ratio = 0.92; 95% Confidence Interval = 0.52–1.63; *p* = 0.884). In conclusion, we did not find evidence of an association between A380T (*ITGα6*) and the risk of developing breast cancer. The results represent the first report of these genetic variations in breast cancer; therefore, they are an important contribution to the literature.

## 1. Introduction

Breast cancer is the leading cause of cancer deaths in women. There are 18,000 new cases per year, representing 17.8% of total cancer incidence in Argentina. However, there is a 90% chance of healing in cases of early detection which is an essential tool, together with prevention, to stop the progression of the disease [1].

Integrins are heterodimeric receptors that consist of paired α and β subunits. In the human genome, there are 18 α and 8 β subunits that combine in a limited combination to provide 24 integrin receptors, each with its own specificity for select extracellular matrix (ECM) or cellular adhesion proteins [2,3]. Integrins have two major functions, adhesive and transduction, which are involved in essential tasks of the cell, such as anchor, migration, survival, proliferation, cell growth and differentiation [3,4].

Integrins containing the α6 subunit are laminin receptors in which the α6 subunit can pair with either the β1 or β4 subunit to form α6β1 and α6β4 integrins, respectively [2,5,6]. The α6β4 integrin is predominantly expressed in epithelial cells where it is present at the basal surface adjacent to the basement membrane and nucleates the formation of hemidesmosomes. These stable adhesions are critical for the integrity of epithelial monolayers. In contrast to this function, integrin α6β4 signaling in various cancers promotes an invasive and metastatic phenotype. This functional change is mediated by phosphorylation of the cytoplasmic tail of the integrin β4 subunit that releases integrin α6β4 from hemidesmosomes and allows the integrin to promote invasive signaling through cooperation with growth factor receptors and alteration of the transcriptome which in turn facilitates tumor progression [5,6,7,8,9,10,11,12].

Integrin subunits (α6 and β4) are encoded by the *ITGα6* and *ITGβ4* genes, respectively [13]. Structurally, the α6β4 integrin differs from others by the β4 cytoplasmic domain, which performs most of integrin functions, and it is much longer than any other β subunit (about 1000 residues). It is arranged in four functional isoforms, a product of alternative splicing [14]. Furthermore, β4 consists of four fibronectin type III domains (FnIII). The association between β4 and the cytoskeleton (keratin intermediate filaments) is mediated by plectin, and it would be possible that hemidesmosome dissociation is influenced by inhibition of the α6β4-plectin interaction, particularly in β4 FnIII 1 and 2 domains. The β4-plectin interaction could be allosterically regulated by β4 conformation and mutagenic analyses also revealed that these sites are hot spots [15]. Furthermore, the intracellular portion of α6 has a short tail and it is found in two isoforms with no functional differences [14].

The α6β4 integrin appears to have importance in breast cancer malignancy, as well as in other epithelial carcinomas [12,13,14,16]. There is strong evidence that α6β4 expression is pathologically significant [17]. However, the genetic determinants involved in susceptibility to breast cancer have not yet been fully studied.

Genetic variations in low penetrance genes could have a significant impact on the breast cancer etiology [18]. Thus, single nucleotide polymorphisms (SNPs) or point mutations in functionally relevant gene regions of the α6β4 integrin could promote breast cancer development as well as tumor progression and metastasis. The discovery of new genetic markers may contribute to the identification of differentially susceptible individuals to breast cancer and could have importance as potential therapeutic targets.

In this context, previous studies have identified a pathogenic mutation in the 3841 nucleotide position of the *ITGβ4* gene that results in an amino acid change in the 1281 position of the β4 subunit, R1281W (rs121912467) [19]. This residue is located in FNIII2 domain and is critical for the interaction with plectin. In consequence, the binding site undergoes changes, affecting the structure of the hemidesmosomes and decreasing cell adhesiveness, which are requirements for carcinogenic development [15,20,21]. On the other hand, a high frequency of the α6 subunit A380T polymorphism (rs11895564) has been observed in papillary thyroid carcinoma. A380T was mainly associated with the size, number and tumor lymphatic metastasis [22].

However, neither A380T (*ITGα6*) nor R1281W (*ITGβ4*) have been previously examined in breast cancer. Therefore, in order to investigate the potential role of genetic variations (A380T and R1281W) in breast cancer susceptibility, we performed a case-control study in a female population from the northeast region of Argentina (Posadas, Argentina).

## 2. Results

### 2.1. Genotyping

**Figure 1:**
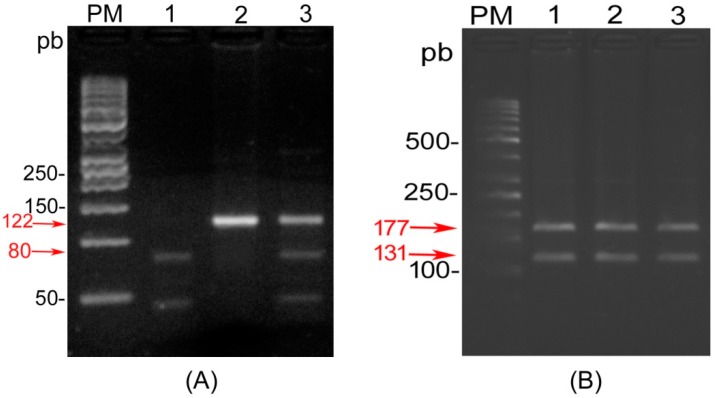
RFLP analysis in 3% agarose gel. (**A**) Enzymatic digestion of *ITGα6* (A380T) SNP with TaaI, lane 1: variant genotype (AA); lane 2: wild-type homozygous (GG) and lane 3: heterozygous (GA); (**B**) Enzymatic digestion of *ITGβ4* (R1281W) mutation with BsrI, lanes 1–3: banding prototype for wild-type genotype (CC). PM: molecular weight of 50 bp.

**Figure 2:**
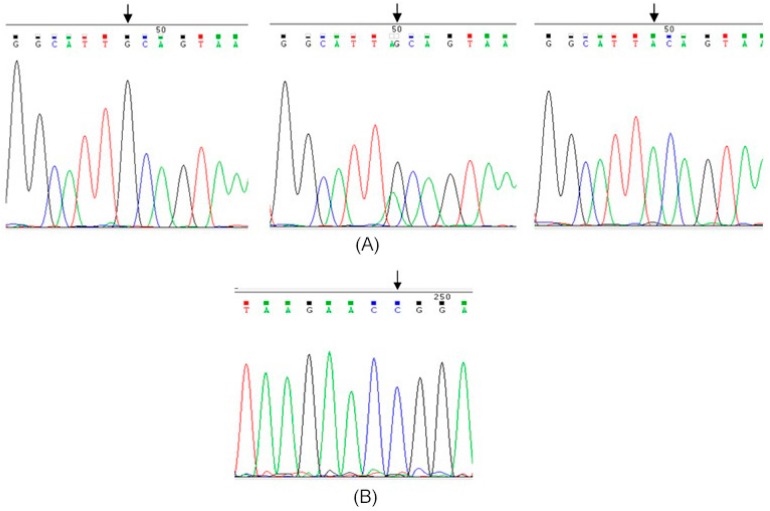
Sequencing results. (**A**) Genotype patterns for *ITGα6* (A380T) loci, wild-type GG (**left**); heterozygous GA (**middle**) and variant genotype AA (**right**); (**B**) Wild-type pattern for *ITGβ4* (R1281W) loci. Arrows indicate the variant position.

Restriction analysis of the *ITGα6* (A380T) and *ITGβ4* (R1281W) genetic variants is shown in Figure 1, respectively. Furthermore, in order to confirm the genotype pattern, selected samples which represented different banding prototypes were submitted for sequencing (Figure 2).

### 2.2. Genotypic/Allelic Frequency and Association Analysis

For the *ITGβ4* (R1281W) mutation, we found that all individuals analyzed in both cases and control groups were homozygous for the wild-type genotype CC (Arg). No variant allele carrier (Arg/Trp or Trp/Trp) was found in the population studied; therefore, association analysis was not performed.

Otherwise, allelic and genotype frequencies of the *ITGα6* (A380T) genetic variant in patients and controls are given in Table 1. Distribution of the A380T genotypes revealed no significant deviations from Hardy-Weinberg equilibrium among cases (χ^2^ = 0.892; *p* = 0.34) and the control group (χ^2^ = 0.776; *p* = 0.38). According to the codominant model, the prevalence of the variant genotype (AA) was 8% and 9% in cases and controls, respectively. A high frequency of heterozygous genotype GA in cases compared to controls was observed, achieving values of 48% and 49%, respectively.

Regarding the *ITGα6* (A380T) SNP association analysis, our data showed that individuals in the homozygous state AA (Thr/Thr) or heterozygous GA (Ala/Thr) (dominant model: Odds Ratio (OR) = 0.92; 95% Confidence Interval (CI) = 0.52–1.63; *p* = 0.884) are not significantly associated with breast cancer development; furthermore, no inheritance model showed a different result (Table 1). The distribution of variant allele A (Thr) in cases and controls reached values of 32% and 34%, respectively. The risk in patients carrying the A (Thr) allele is not significant (OR = 0.92; 95% CI = 0.61–1.41; *p* = 0.747) (Table 2).

#### Stratification Analysis

The OR adjusted by clinical-pathological parameters in breast cancer patients showed no differences among estimated strata risks; therefore, there is no association between variant genotypes (GA + AA) with breast cancer risk (Table 3).

## 3. Discussion

A380T is a genetic variant located on exon 7 of the *ITGα6* gene and it produces a change of the small and non-polar amino acid Alanine (Ala) to another small but still polar amino acid Threonine (Thr) in the intracellular region of the α6 subunit. Structurally, the small size of both amino acids could not have significant changes in the protein. Nevertheless, the differences in their electric charge could affect normal hydrophobic interactions due to the presence of the polar groups of the new residue. This could also alter interactions between the α6 and β4 subunits of the integrin.

Previous studies have found an association between the *ITGα6* (380Thr, rs11895564) SNP and papillary thyroid carcinoma and intra-cerebral hemorrhage development [22,23].

However, in our study, allelic frequencies of the *ITGα6* (380Thr) genetic variant in cases and controls were similar, reaching values of 32% and 34%, respectively. The association analysis showed that there is no association between the A380T (G>A) SNP and susceptibility to breast cancer development. Results of all inheritance models were coincident and showed values close to the null association (OR = 1), supporting the lack of association. Furthermore, stratification analysis by the clinical-pathological parameters in patients indicated that there is no association between the variant genotypes Ala380Thr (GA and AA) and breast cancer risk, showing no differences in estimated risks among strata.

Moreover, the R1281W mutation in exon 31 of the *ITGβ4* gene causes a residue substitution of Arginine positively charged by another aromatic or non-polar Tryptophan. These amino acids are both large and this substitution could also involve alterations in normal polar interactions by the incorporation of the aromatic group of the new residue.

In addition, this mutation lies in the FNIII2 domain of the β4 integrin and therefore it might be altering the interaction with plectin, disrupting hemidesmosome integrity and promoting cell migration, a requirement for metastatic development [15,20,21].

Although this mutation has been linked to nonlethal forms of epidermolysis bullosa [19], there was no data until now from other diseases, making this work the first antecedent in breast cancer. In this work, the frequency of the *ITGβ4* (R1281W) mutation was null in the subjects studied.

## 4. Materials and Methods

### 4.1. Study Subjects

We evaluated the *ITGα6* codon 380 and *ITGβ4* codon 1281 polymorphisms in 85 breast cancer patients (mean age: 55.7 ± 12.25) and 113 control subjects whose age group matched the case. Clinical-pathological parameters of breast cancer patients were gathered from ascertainment of medical records (Table 4). All individuals came from the same region of Argentina (Misiones). The studies obtained proper approval from the Local Ethical Committee of Dr. Ramon Madariaga Hospital of Posadas, Misiones.

### 4.2. PCR-RFLP Analysis

Blood samples were collected and DNA was extracted by salting out method [24]. A380T SNP (*ITGα6*) and R1281W mutation (*ITGβ4*) were analyzed by using Polymerase Chain Reaction-Restriction Fragment Length Polymorphism (PCR-RFLP).

Primers used for SNPs region amplification were designed with Primer-BLAST tool and their sequences were as follows: *ITGα6* (F: 5′-ACATGAACCAGCAAGGCAGA-3′; R: 5′-ACCTGGGTAGCCATCTTGATT-3′) and *ITGβ4* (F: 5′-TATTGGGCCCATGAAGAAAG-3′; R: 5′-CCACGATAGGGATGTCAGGG3′), giving products size of 122 and 298 pb, respectively [25].

Each PCR amplification was performed in a total volume of 20 µL containing 1× PCR buffer (KCl (pH 8.8)), 1.5 mM MgCl_2_, 100 µM of each dNTP, 5 pmol of each primer, 0.5 U Taq polymerase (Thermo Scientific) and 1 μL of the DNA template (100 ng/μL).The cycling conditions started with an initial denaturation at 94 °C for 3 min followed by 30 cycles of denaturation at 94 °C for 30 s, hybridization at 58 °C (*ITGα6*) and 61 °C (*ITGβ4*) for 30 s, extension at 72 °C for 30 s and a final extension of 72 °C for 5 min. All reactions were performed in a BIOER GenePro Thermocycler.

PCR products were subjected to electrophoresis on 2% agarose gel stained with GelRed™ (Nucleic Acid Gel Stain 10,000× in water) and visualized under UV light for the control of their specificity and integrity. Product sizes were confirmed by comparison with a 100 bp DNA ladder (GenBiotech, Buenos Aires, Argentina).

For RFLP analysis, 5 µL of *ITGα6* (122 pb) products were digested with *TaaI* restriction enzyme (Thermo Scientific) overnight at 65 °C. *TaaI* digestion gave fragments of 122 pb for the wild-type genotype (GG), 80 and 42 pb for variant genotype (AA) and 122, 80 and 42 pb for the heterozygous (GA). Likewise, 5 µL of *ITGβ4* (298 pb) products were digested with *BsrI* restriction enzyme (Thermo Scientific) overnight at 37 °C; giving fragments of 176 and 121 pb for the wild-type genotype (CC), 176, 131, 121 and 46 pb for variant genotype (TT) and 131, 121 and 46 pb for the heterozygous (CT).

Digestion products were separated by electrophoresis on 3% agarose gel stained with GelRed™ (Nucleic Acid Gel Stain 10,000× in water) and visualized under UV light. Different banding patterns obtained were confirmed by direct sequencing.

### 4.3. Statistical Analysis

The Hardy-Weinberg distribution of genotypes and alleles between case and control groups were evaluated using the Chi-squared (χ^2^). Odds ratios (ORs) and confidence intervals (CI) of 95% were used to determine association between *ITGα6* (A380T) and *ITGβ4* (R1281W) with susceptibility of breast cancer development. The risk associated with each genotype was determined according to the different models of inheritance and a *p* value <0.05 was considered statistically significant. This analysis was performed using EPIDAT 3.1 (Program Analysis for Epidemiologic Data Tabulations; Xunta de Galicia, Coruña, España—Pan American Health Organization, Washington, DC, USA).

## 5. Conclusions

In this work, we found that the A380T SNP of the *ITGα6* gene was not associated with breast cancer development in the analyzed population of Misiones, Argentina. No subjects carrying the R1281W mutation in the *ITGβ4* gene were found and this absence could reflect its high deleterious impact on proteins, so it would be eliminated from the population by natural selection. Moreover, to more precisely determine the contribution of these genetic variants on *ITG*α*6β4* to breast cancer incidence, further investigation in other populations is needed.

These results represent the first report of these genetic variations in breast cancer; therefore, they are an important contribution to the literature.

## Figures and Tables

**Table 1:** Risk analysis of *ITGα6* (A380T) SNP (G>A) based on inheritance models.

Models ^1^	Genotype ^2^	Cases		Controls		Odds Ratio (OR)	Confidence Interval (CI) 95%	*p* ^4^
		*n* = 85	(%) ^3^	*n* = 113	(%) ^3^			
Co	GG	37	44	47	42	1	-	-
	GA	41	48	55	49	0.94	0.52–1.71	0.881
	GG	7	8	11	9	0.81	0.28–2.29	0.795
Do	GG	37	44	47	42	1	-	-
	GA + AA	48	56	66	58	0.92	0.52–1.63	0.884
Re	GG + GA	78	92	102	91	1	-	-
	AA	7	8	11	9	0.83	0.31–2.24	0.806
Ad	-	-	-	-	-	0.92	0.61–1.41	0.747

^1^ Inheritance models: codominant (Co), dominant (Do), recessive (Re), additive (Ad); ^2^ Genotypes and their groups for G>A polymorphism; ^3^ Genotype frequencies expressed in percentage; ^4^ Fisher exact test.

**Table 2:** Allele distribution of *ITGα6* (Ala380Thr) SNP in breast cancer patients and control subjects.

*ITGα6* Codon 380	Cases *n* = 85 (%)	Controls *n* = 113 (%)	OR	CI 95%	*p* ^1^
Allelic Frequencies					
Ala	115 (68)	149 (66)	0.92 ^2^	0.61–1.41 ^2^	0.747 ^2^
Thr	55 (32)	77 (34)			

^1^ Fisher exact test; ^2^ Thr allele vs. Ala allele.

**Table 3:** Stratification analysis by clinical-pathological parameters in breast cancer patients.

Parameters	GA + AA Cases/Controls ^1^	GG Cases/Controls ^2^	OR Adjusted (95% CI)	*p* ^3^
Age				
<55 (mean)	21	17	1.13 (0.55–2.31)	0.857
≥55 (mean)	23	18	0.91 (0.44–1.87)	0.854
Tumor site				
Ductal	21	13	1.15 (0.52–2.52)	0.843
Lobular	1	-	-	1.000
Lymphovascular invasion				
Positive	24	16	1.07 (0.51–2.22)	1.000
Negative	13	12	0.77 (0.32–1.84)	0.656
RP				
Positive	21	13	1.15 (0.52–2.52)	0.843
Negative	6	5	0.85 (0.25–2.96)	1.000
RE				
Positive	22	13	1.21 (0.55–2.63)	0.697
Negative	9	3	2.13 (0.55–8.32)	0.359
HER2				
Positive	10	9	0.79 (0.29–2.09)	0.803
Negative	20	8	1.78 (0.72–4.38)	0.280
Histological grade				
Grade I	4	4	0.71 (0.17–2.99)	0.720
Grade II	26	18	1.02 (0.51–2.08)	1.000
Grade III	13	10	0.92 (0.37–2.28)	1.000
Menopausal period				
Premenopausal	12	14	0.61 (0.26–1.44)	0.280
Postmenopausal	24	17	1.01 (0.48–2.07)	1.000

^1^ Controls: GA + AA = 66; ^2^ Controls: GG = 47; ^3^ Adjustment obtained in logistic regression models.

**Table 4:** Clinical-pathological parameters of breast cancer patients.

Parameters	Total Number of Cases (*n* = 85)
Pathological diagnosis	
Ductal carcinoma	36
Lobular carcinoma	1
N/D ^1^	48
Lymphovascular invasion	
Positive	43
Negative	30
N/D	14
Histological grade	
Grade I	8
Grade II	45
Grade III	26
N/D	8
Menopausal period	
Premenopausal	29
Postmenopausal	45
N/D	13
Estrogen receptor (ER)	
Positive	32
Negative	17
N/D	38
Progesterone Receptor (PR)	
Positive	37
Negative	12
N/D	38
HER2/neu	
Positive	21
Negative	28
N/D	38

^1^ N/D: No data.
